# Efficacy and safety of bupleurum-containing formulas combined with chemotherapy for triple-negative breast cancer: a systematic review and meta-analysis

**DOI:** 10.3389/fonc.2026.1867349

**Published:** 2026-07-15

**Authors:** Shangshang Li, Jie Chen, Longzhu Ke, Yuanxiu Leng, Guanglai Cui, Qianyu He, Hong Chen, Ping Jiang, Li Luo

**Affiliations:** 1 Guizhou University of Traditional Chinese Medicine, Guiyang, China; 2 Traditional Chinese Medicine Rehabilitation Department, Guihang Pingba Hospital, Anshun, China; 3 College of Traditional Chinese Medicine, Hubei University of Chinese Medicine, Wuhan, China; 4 Department of Oncology, GuiHang Guiyang Hospital, Guiyang, China; 5 Hospital Office of the President, Guihang Guiyang Hospital, Guiyang, China

**Keywords:** bupleurum, chemotherapy, meta-analysis, traditional Chinese medicine, triple-negative breast cancer (TNBC)

## Abstract

**Background:**

Bupleurum-containing formulas are widely used in China as adjunct to chemotherapy for triple-negative breast cancer (TNBC). However, systematic evidence on their efficacy and safety remains limited. This study evaluates the clinical effects and safety of Bupleurum-containing formulas combined with chemotherapy in the patients with TNBC.

**Methods:**

We searched seven electronic databases (PubMed, EMBASE, Cochrane Library, China National Knowledge Infrastructure (CNKI), Sinomed, VIP Database, and Wanfang Database) from inception to January 31, 2026. Studies meeting predefined inclusion criteria were identified through a systematic search. Two researchers independently conducted study selection, data extraction, and risk of bias assessment to ensure accuracy and reduce potential bias.

**Results:**

Compared with chemotherapy alone, Bupleurum-containing traditional Chinese medicine (TCM) formulas combined with chemotherapy improved several clinical outcomes in patients with TNBC. The combined treatment significantly increased the objective response rate (ORR) (RR = 1.32, 95% CI: 1.18–1.48, *p* < 0.00001) and disease control rate (DCR) (RR = 1.15, 95% CI: 1.09–1.21, *p* < 0.00001), and improved quality of life (QOL) (MD = 6.48, 95% CI: 4.93–8.03, *p* < 0.00001). It was also associated with changes in peripheral blood CD3^+^, CD4^+^, CD8^+^ T lymphocytes, as well as the CD4^+^/CD8^+^ ratio. Subgroup analysis indicated that interventions lasting ≥12 weeks was associated with greater improvements in ORR. In terms of safety, the combined regimen reduced the incidence of grade III-IV gastrointestinal adverse events.

**Conclusion:**

Combining Bupleurum-containing TCM formulas with chemotherapy shows potential in improving ORR, DCR, and QOL, while reducing treatment-related toxicity in patients with TNBC. This approach may serve as an adjunctive strategy, but these findings should be interpreted with caution, and further high-quality studies are needed.

This systematic review and meta-analysis followed the Preferred Reporting Items for Systematic Reviews and Meta-Analyses (PRISMA) guidelines. The protocol was prospectively registered in the PROSPERO database (registration number: CRD420251269445).

**Systematic review registration:**

https://www.crd.york.ac.uk/prospero/, identifier CRD420251269445.

## 1 Introduction

By 2024, breast cancer has become the most common malignant tumor among women worldwide and remains the leading cause of cancer-related death in this population (1). Triple-negative breast cancer (TNBC), which accounts for approximately 15%–20% of cases, is defined by the absence of estrogen receptor (ER), progesterone receptor (PR), and human epidermal growth factor receptor 2 (HER2) expression. This subtype is characterized by high invasiveness, marked heterogeneity, and a tendency for early visceral metastasis, and is associated with a poorer prognosis than other breast cancer subtypes (2).

For early-stage TNBC, treatment typically involves surgical resection combined with neoadjuvant chemotherapy, radiotherapy, and other multimodal approaches. Neoadjuvant chemotherapy can reduce tumor burden, improves resectability, and increase pathological complete response (pCR) rates, thereby improving outcomes. In advanced TNBC, palliative chemotherapy remains the main approach, with the goal of controlling disease progression and prolonging survival (3). However, commonly used regimens (primarily platinum-, taxane-, and anthracycline-based therapies) are associated with substantial toxicities. These include dose-limiting toxicities (DLTs) such as myelosuppression and severe gastrointestinal reactions. Such adverse effects not only reduce quality of life (QOL) but can also lead to dose reductions or treatment interruptions, which may compromise treatment effectiveness and survival (4).

In recent years, the introduction of new agents—including immune checkpoint inhibitors such as Pembrolizumab (5), PARP inhibitors such as Olaparib (6), and antibody-drug conjugates such as Sacituzumab Govitecan (7)—has expanded treatment options for TNBC. However, these advances have not eliminated chemotherapy-related toxicities. Identifying adjuvant therapies that can improve efficacy while reducing toxicities remains an important focus in TNBC research.

Traditional Chinese medicine (TCM) has been used as an adjunct in cancer treatment, particularly in efforts to reduce toxicity and improve tolerance to therapy (8). In TCM theory, breast cancer is often associated with “liver *Qi* stagnation, ” which can lead “*Qi* stagnation and blood stasis” sometimes interpreted as reflecting dysregulation of the tumor microenvironment (TME) (9). The therapeutic approach of “soothing the liver and regulating *Qi*” is therefore used to address both symptoms and underlying imbalance, and may also influence the TME.

Bupleurum is a commonly used herb in TCM for “soothing the liver and regulating *Qi*, ”, and is frequently used in formulas used in breast cancer care (10). Its main active components, such as saikosaponins, have been reported to inhibit TNBC progression by suppressing tumor metastasis and reversing multidrug resistance (11–13). Although several clinical studies have examined Bupleurum-containing formulas combined with chemotherapy in TNBC, the findings are scattered and lack synthesis. This study therefore conducts a meta-analysis to evaluate the efficacy and safety of this combined approach, the aim of providing evidence to inform clinical practice.

## 2 Methods

This systematic review and meta-analysis followed the Preferred Reporting Items for Systematic Reviews and Meta-Analyses (PRISMA) guidelines. The protocol was prospectively registered in the PROSPERO database (registration number: CRD420251269445).

### 2.1 Search strategy

A systematic search was conducted across seven electronic databases, including PubMed, EMBASE, the Cochrane Library, China National Knowledge Infrastructure (CNKI), Sinomed, VIP Database, and Wanfang Database, to identify studies published up to January 31, 2026. The search combined Medical Subject Headings (MeSH) and free-text terms related to “triple-negative breast cancer, ” “triple-negative breast neoplasms, ” “chemotherapy, ” “chemical drug therapy, ” “Bupleurum, ” “soothing the liver and relieving depression, ”, and “regulating *Qi*.” The full search strategies for each database are provided in the **Supplementary Material**. The search was limited to studies published in English or Chinese.

### 2.2 Inclusion and exclusion criteria

Studies were included if they met the following criteria: (1) randomized controlled trials (RCTs); (2) participants diagnosed with TNBC based on histopathological or cytological criteria; (3) the intervention group received Bupleurum-containing TCM formulas combined with chemotherapy, while the control group received the same chemotherapy regimen alone or chemotherapy with placebo; (4) at least one of the following outcomes was reported: tumor response, QOL, adverse events (AEs), or peripheral blood lymphocyte counts. Studies were excluded if they met any of the following criteria: (1) non-RCT study designs (e.g., observational studies, case reports); (2) participants with severe infections, other malignancies, or life-threatening comorbidities; (3) insufficient or unavailable data for extraction; (4) baseline characteristics that were not comparable between groups; (5) duplicate publications from the same cohort, in which case only the most complete or most recent report was included.

### 2.3 Study selection and data extraction

Study selection and data extraction were performed independently by two researchers according to the predefined inclusion and exclusion criteria. Disagreements were resolved through discussion, with a third researcher consulted when needed. The following information was extracted from each study: study characteristics (first author and publication year), patient characteristics (sample size and age), intervention details (treatment regimens and duration), and reported outcome along with their assessment methods.

### 2.4 Risk of bias assessment

The risk of the included studies was assessed using the Cochrane Risk of Bias Tool. Two researchers conducted the assessment independently, and disagreements were resolved through discussion. The tool evaluates seven domains, which we grouped into three categories for clarity: (1) selection bias (random sequence generation and allocation concealment); (2) performance and detection bias (blinding for participants, personnel, and outcome assessors); and (3) attrition and reporting bias (incomplete outcome data, selective reporting, and other sources of bias). Each domain was rated as low risk, high risk, or unclear risk according to established criteria.

### 2.5 Outcome definitions

The primary outcomes were tumor response and QOL. Tumor response was assessed using the ORR and DCR. Disease status, including complete response (CR), partial response (PR), stable disease (SD), and progressive disease (PD), was defined according to World Health Organization (WHO) criteria (14) or the Response Evaluation Criteria in Solid Tumors (RECIST) (15). QOL was evaluated using the Karnofsky Performance Status (KPS) scale (16).

Secondary outcomes included 1-year survival rate, adverse events (AEs), tumor markers levels (CEA, CA125, and CA15-3), and peripheral blood lymphocyte counts. AEs were assessed according to WHO criteria (14) or the Common Terminology Criteria for Adverse Events (CTCAE) (17), with a focus on hematological toxicities (e.g., myelosuppression, leukopenia, neutropenia, hemoglobin reduction, and thrombocytopenia), gastrointestinal toxicities (e.g., nausea, vomiting, diarrhea, and overall gastrointestinal reactions), and alopecia. Peripheral blood lymphocyte were evaluated by measuring T cell subsets (CD3^+^, CD4^+^, and CD8^+^ T cells) and the CD4^+^/CD8^+^ ratio.

### 2.6 Statistical Analysis

Data analysis was performed using Review Manager (RevMan) version 5.4. A random-effects model was used to pool effect sizes. For dichotomous outcomes, results were expressed as risk ratios (RRs), and for continuous outcomes, as mean differences (MDs), both with 95% confidence intervals (CIs). Heterogeneity was assessed using the *Chi*-square test and the I² statistic, with *p* < 0.05 considered statistically significant.

When an outcome was reported in 10 or more studies, publication bias was assessed using funnel plots and Egger’s test. Subgroup analyses were conducted to explore potential sources of heterogeneity, particularly for tumor response outcomes such as ORR and DCR.

### 2.7 Quality assessment of evidence

The quality of evidence was evaluated using the GRADE (Grading of Recommendations Assessment, Development and Evaluation) approach (18). Evidence was classified as high, moderate, low, and very low quality. Ratings were downgraded based on the following factors: risk of bias, inconsistency across studies (e.g., I² > 50% or *p* < 0.10 for the *Chi*-square test), indirectness, imprecision (e.g., wide CI or small sample sizes), and potential publication bias. Reasons for downgrading were documented in the footnotes of the evidence summary tables.

## 3 Results

### 3.1 Search results

**Figure 1:**
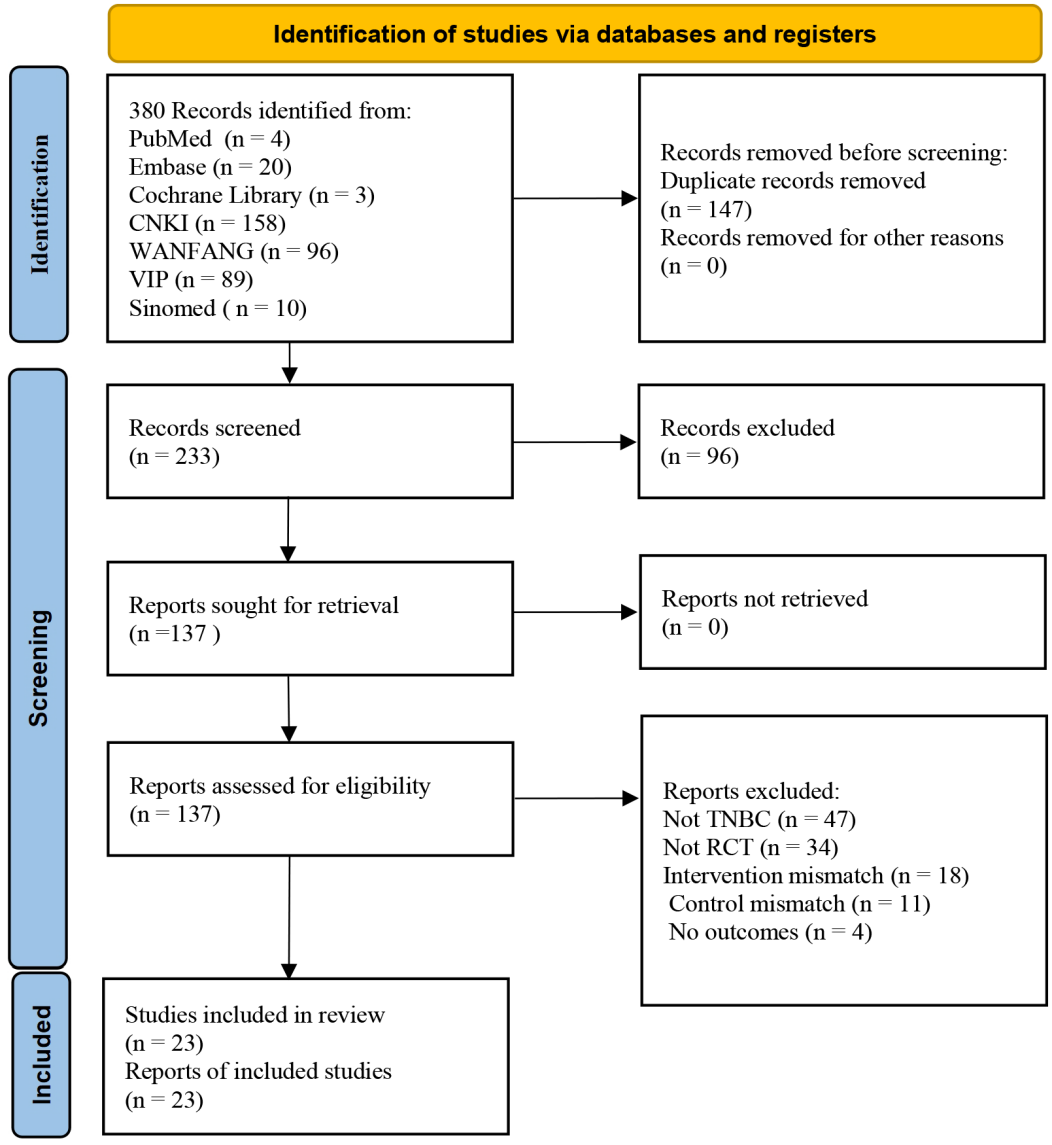
Flow diagram of the selection process.

A total of 380 records were identified from Chinese and English databases. After removing 147 duplicates, 233 records remained for screening. Following title and abstract review, 96 studies were excluded. The remaining articles underwent full-text assessment, after which 114 studies were excluded based on the predefined criteria. In total, 23 studies were included in the analysis, comprising 1, 680 patients with TNBC (**Figure 1**) (19–41).

### 3.2 Study characteristics

The characteristics of the included studies are summarized in **Table 1**. All 23 studies were conducted in China and published between 2014 and 2025, including 1, 680 female patients with pathologically confirmed TNBC. Sample sizes ranged from 46 to 137. In all studies, the control group received conventional chemotherapy, while the experimental group received chemotherapy combined with Bupleurum-containing TCM formula. Tumor responses were assessed using RECIST criteria in 14 studies and WHO criteria in 5 studies. A total of 16 studies reported adverse events. Among these, five used the NCI-CTCAE criteria for toxicity grading, two used WHO criteria, and nine did not specify the assessment method. The compositions of the Bupleurum-containing TCM formula are provided in **Supplementary Table 1**.

**Table 1:** Baseline characteristics of the included studies.

Study ID	Criteria	Sample size (T/C)	TNM stage	Treatment group	Control group	Duration	Outcome
Cui. 2015	WHO	30/30	I-III	Bupleurum-based Formula + AT	AT	8w	O1, O2
He et al., 2021	WHO/Un	28/28	I-III	Cigu Pingyan Decoction + AC-T	Doc+Cyc	24w	O1, O2, O3, O8
Jiang et al., 2022	Un	62/62	II-III	Shugan Yishen Formula + Tax+Epi	Tax+Epi	12w	O3, O4, O8, O9
Ju. 2021	Un	36/36	IV	Shugan Jianpi Formula + GP	GP	6w	O3, O8, O9
Li et al., 2019	RECIST/Un	31/31	IV	Bupleurum-based formula + Tax+Gem	Tax+Gem	18w	O1, O2, O7, O8
Li. 2014	RECIST	26/20	IV	Bupleurum-based Formula + GP	GP	18w	O1, O2, O3, O4
Lin. 2016	RECIST	32/33	IV	Bupleurum-based Formula + CEF	CEF	12w	O1, O2, O5
Liu. 2025	RECIST	46/46	II-III	Shugan Jieyu Decoction + TC	TC	18w	O1, O2, O6, O7
Mao et al., 2017	RECIST/Un	34/34	IV	Fuzheng Hualiu Formula + GP	GP	6w	O1, O2, O3, O6, O8
Ren. 2016	WHO/Un	23/23	IV	Bupleurum-based Formula + Tax+Gem	Tax+Gem	15w	O1, O2, O8
Song. 2018	/	36/36	IV	Shugan Huazheng Capsule + TAC	TAC/EC	6w	O3, O6
Tian et al., 2022	RECIST	30/30	IV	Shugan Liqi Xiaoyan Formula + EC	EC	12w	O1, O2, O3, O6
Wang et al., 2020	RECIST/NCI	32/31	IV	Shugan Jianpi Formula + GP	GP	6w	O1, O2, O3, O8, O9
Wang. 2015	RECIST/NCI	39/40	IV	Bupleurum-based Formula + Tax+Gem	Tax+Gem	18w	O1, O2, O5, O8, O9
Wang. 2022	WHO/Un	25/25	IV	Shugan Jianpi Yishen Decoction + Tax+Gem	Tax+Gem	Un	O1, O2, O8, O9
Wu et al., 2018	RECIST/Un	31/31	IV	Bupleurum-based Formula + Doc+Epi	Doc+Epi	2 months	O1, O2, O5, O6, O7, O8, O9
Zhang et al., 2016	WHO	70/67	Un	Pingyu Shiru Formula + AC-T	Doc+Cyc	12w	O9
Zhang et al., 2018	RECIST/NCI	25/25	IV	Tiaopi Shugan Yishen Formula + EC	EC	24w	O1, O2, O7, O8, O9
Zhang. 2015	RECIST/NCI	25/25	IV	Bupleurum-based Formula + TC	TC	15w	O1, O2, O8, O9
Zhang. 2022	RECIST/NCI	41/41	I-III	Xiaoyao Loubei Formula + AT	AT	12w	O1, O2, O6, O8, O9
Zhao et al., 2020	RECIST	45/45	IV	Shugan Jianpi Yishen Decoction + Chemo	Chemo	18w	O1, O2, O8, O9
Zhao et al., 2021	WHO/WHO	51/51	I-III	Shugan Jianpi Jiangni Formula + TEC/EC	TEC/EC	18w	O1, O2, O6, O7, O8, O9
Zhu. 2014	RECIST	46/46	IV	Xiaoyao San + Cap	Cap	Until Progression	O1, O2, O3, O4

T, treatment group; C, control group; TNM, tumor-node-metastasis; NR, not reported; w, weeks; WHO, World Health Organization; RECIST, Response Evaluation Criteria in Solid Tumors; NCI, National Cancer Institute; Un, unclear. AT, doxorubicin and paclitaxel; AC-T, doxorubicin and cyclophosphamide followed by paclitaxel; Doc, docetaxel; Cyc, cyclophosphamide; Tax, taxanes; Epi, epirubicin; GP, gemcitabine and cisplatin; Gem, gemcitabine; CEF, cyclophosphamide, epirubicin, and fluorouracil; TC, docetaxel and cyclophosphamide; TAC, docetaxel, doxorubicin, and cyclophosphamide; EC, epirubicin and cyclophosphamide; TEC, docetaxel, epirubicin, and cyclophosphamide; Cap, capecitabine. O, outcome; O1, objective response rate (ORR);O2, disease control rate (DCR); O3, quality of life (QOL);O4, 1-year survival rate;O5, progression-free survival (PFS); O6, tumor markers; O7, immune function;O8, hematological toxicities; O9, non-hematological toxicities.

### 3.3 Risk of bias

**Figure 2:**
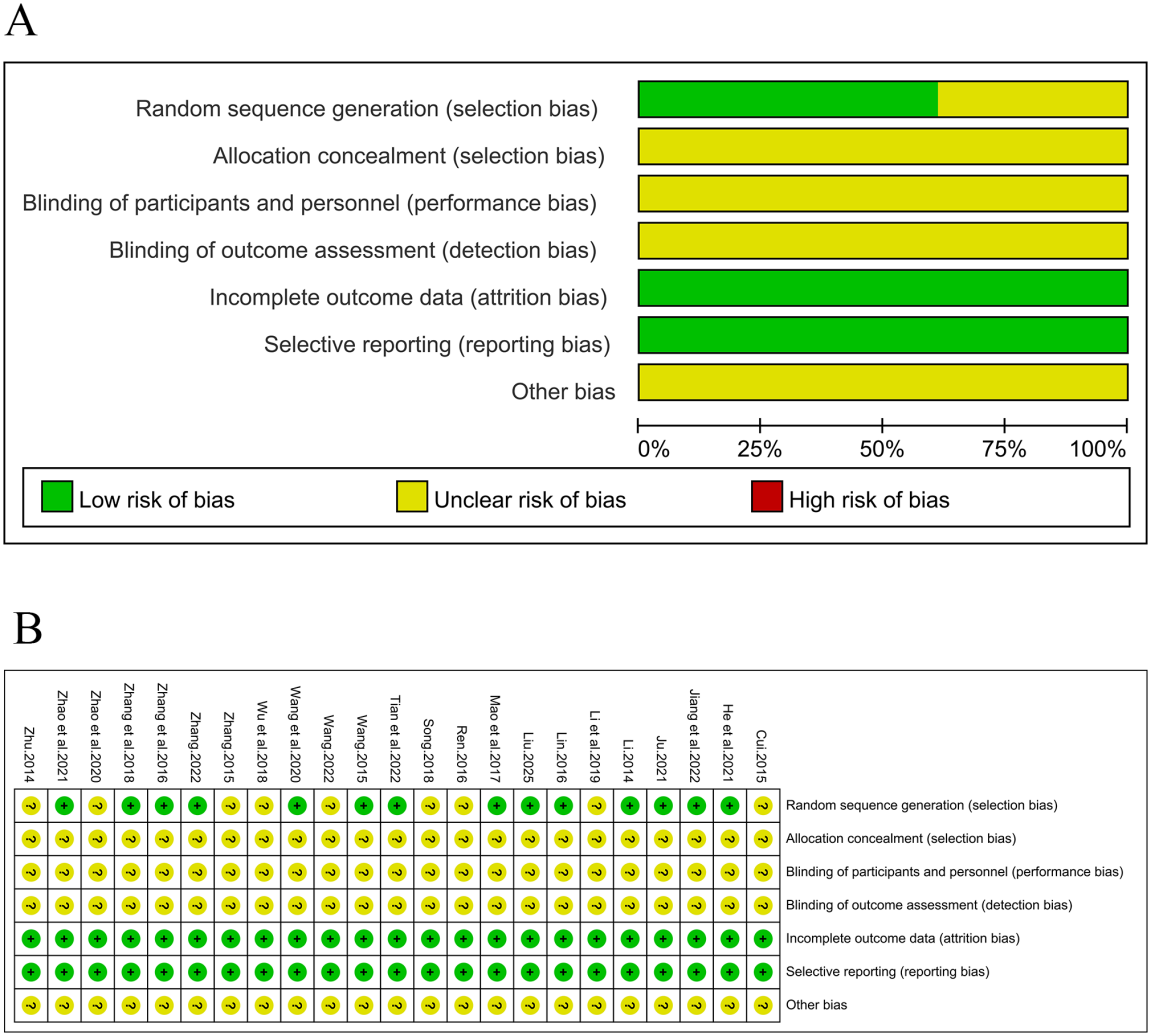
Risk of bias summary and diagram. **(A)** Risk of bias diagram. **(B)** Risk of bias summary.

All 23 studies reported some form of random allocation. Among them, fourteen studies (20–22, 24–27, 30–32, 35, 36, 38, 40) used a random number table for sequence generation and were therefore judged to have a low risk of selection bias. One study assigned patients based on admission order and was considered at high risk of bias. None of the included studies reported details on blinding of participants, personnel, or outcome assessors (**Figure 2**).

### 3.4 Tumor response

**Figure 3:**
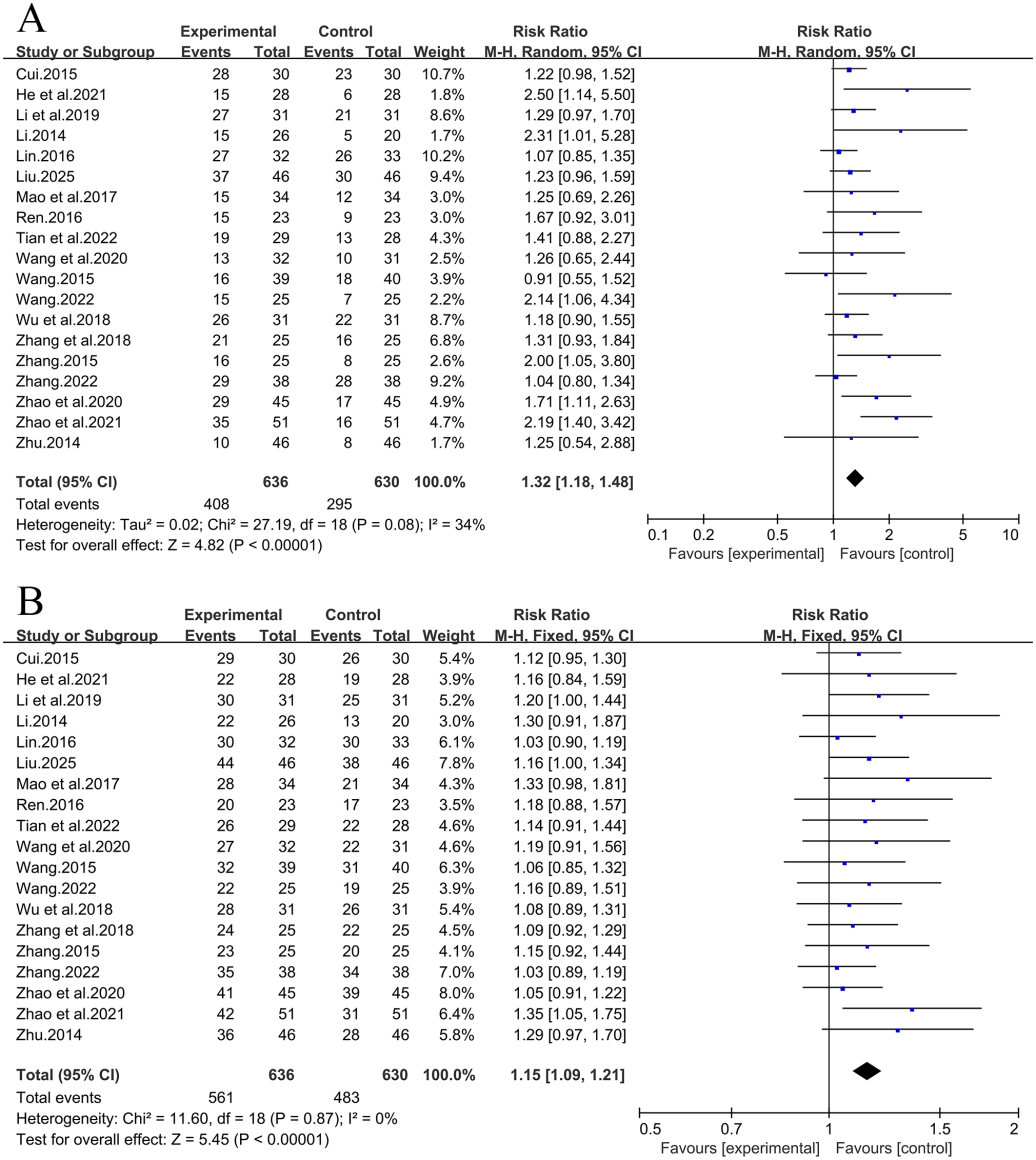
Forest plots of tumor response. **(A)** Forest plot of ORR. **(B)** Forest plot of DCR.

Nineteen trials with 1, 266 participants reported tumor response using WHO or RECIST criteria. For ORR, a random-effects model was applied, yielding a higher ORR in the combination group compared with chemotherapy alone (RR = 1.32, 95% CI: 1.18-1.48, *p* < 0.00001, I² = 34%; **Figure 3A**). For DCR, heterogeneity was low, and a fixed-effects model was used. The results showed a significant improvement in DCR in the combination group (RR = 1.15, 95% CI: 1.09-1.21, *p* < 0.00001, I² = 0%; **Figure 3B**). Overall, these findings indicate that adding Bupleurum-containing formulas to chemotherapy is associated with improved tumor response in patients with TNBC.

### 3.5 Quality of Life

**Figure 4:**
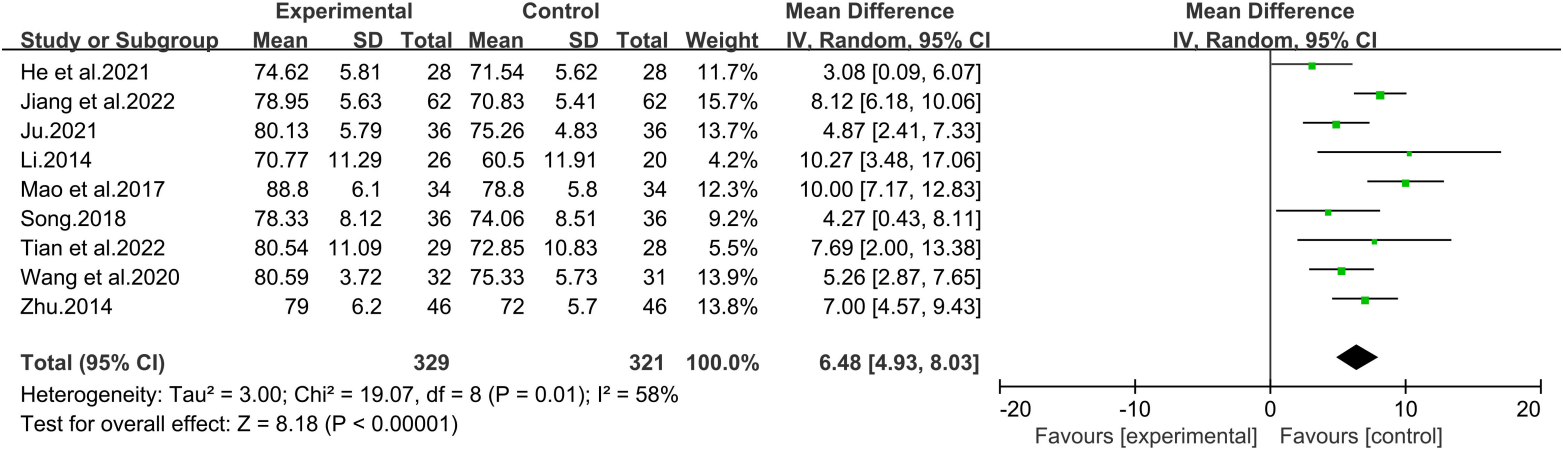
Forest plot of QOL.

Nine trials (*n* = 650) assessed QOL using the KPS scale. A random-effects model showed that the combination therapy significantly improved KPS scores compared with chemotherapy alone (MD = 6.48, 95% CI: 4.93-8.03, *p* < 0.00001, I² = 58%; **Figure 4**).

### 3.6 Survival outcomes

#### 3.6.1 One-year survival rate

**Figure 5:**
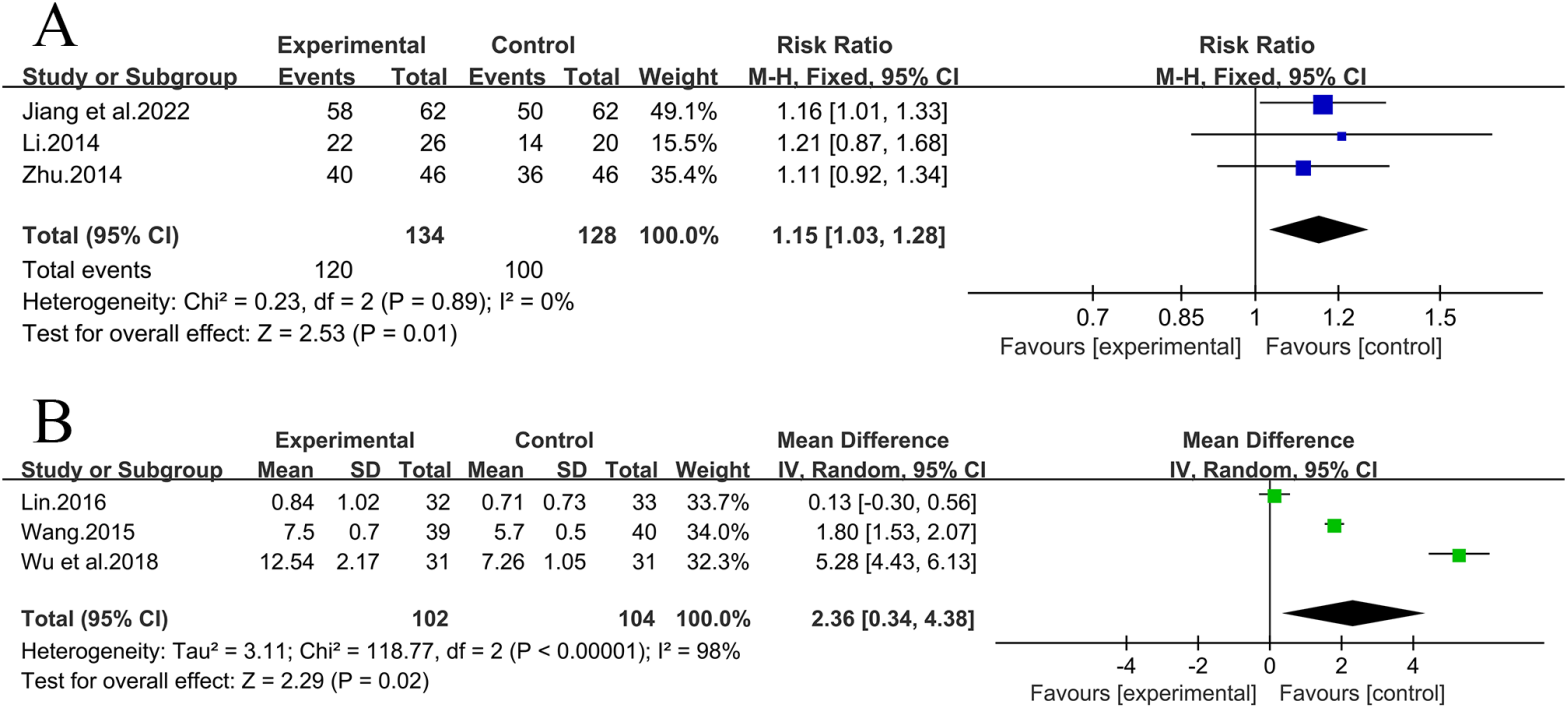
Forest plot of survival outcomes. **(A)** One-year survival rate. **(B)** Progression-free survival (PFS).

Three trials (*n* = 262) reported one-year survival. A fixed-effect model showed a higher one-year survival rate in the combination group compared with chemotherapy alone (RR = 1.15, 95% CI: 1.03-1.28, *p* = 0.01, I² = 0%; **Figure 5A**).

#### 3.6.2 Progression-free survival

Three studies (*n* = 208) provided data on PFS. A random-effects model analysis suggested longer PFS in the combination group (MD = 2.36, 95% CI: 0.34-4.38, *p* = 0.02; **Figure 5B**).

### 3.7 Tumor markers

Compared with chemotherapy alone, the combination therapy was associated with lower levels of tumor markers. Specifically, reductions were observed for carcinoembryonic antigen (CEA) (7 trials; MD = -3.34, 95% CI: -5.49 to -1.19, *p* = 0.002), carbohydrate antigen 125 (CA125) (4 trials; MD = -14.01, 95% CI: -20.71 to -7.31, *p* < 0.0001), and carbohydrate antigen 15-3 (CA15-3) (7 trials; MD = -8.98, 95% CI: -14.24 to -3.73, *p* = 0.0008) (**Supplementary Figure 3**).

### 3.8 Immune function

**Figure 6:**
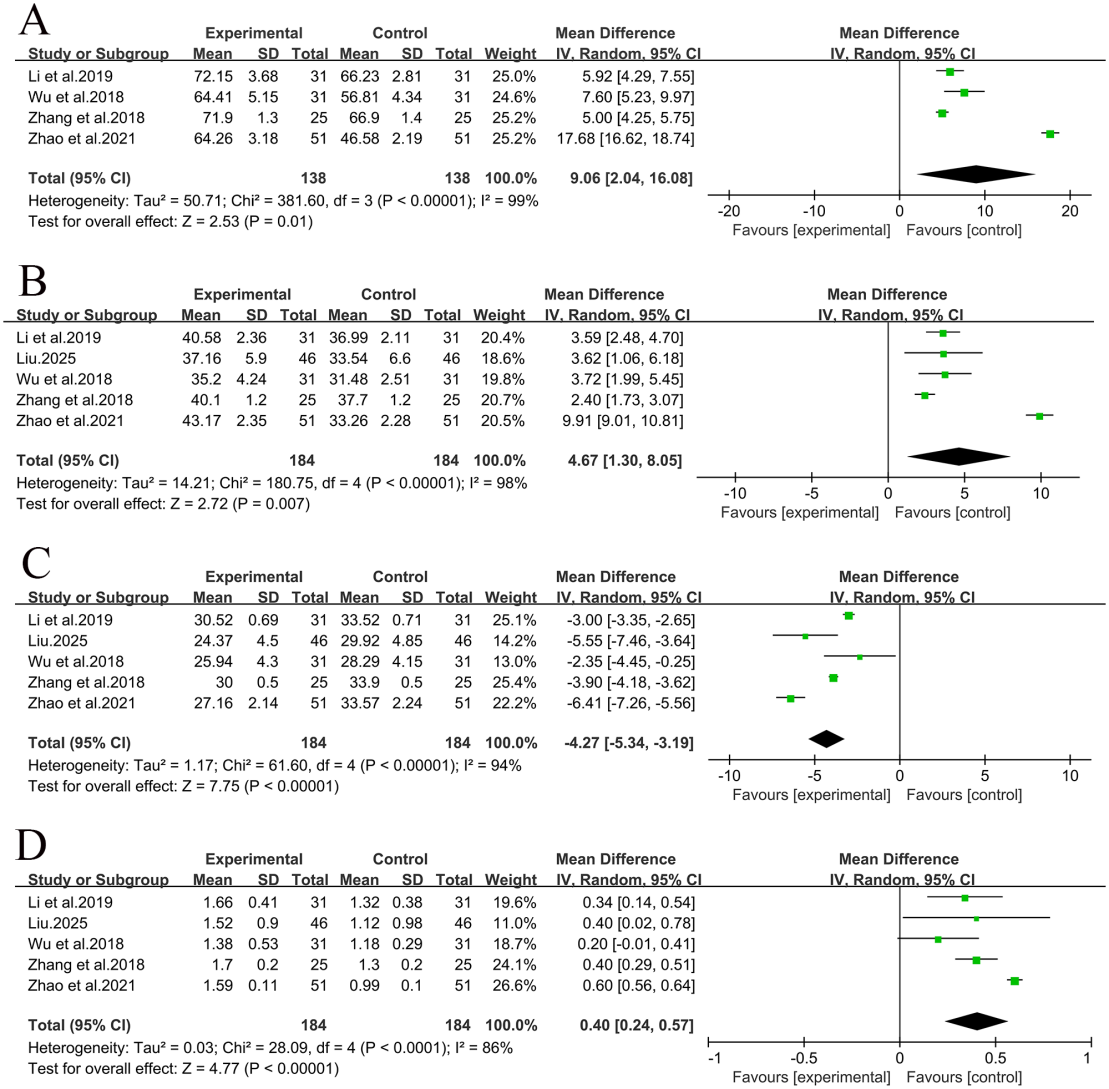
Forest plot of immune function indicators. **(A)** CD3^+^ levels. **(B)** CD4^+^ levels. **(C)** CD8^+^ levels. **(D)** CD4^+^/CD8^+^ ratio.

The combination therapy was associated with changes in T lymphocyte subsets. CD3^+^ T cell levels increased (4 trials; MD = 9.06, 95% CI: 2.04-16.08, *p* = 0.01; **Figure 6A**), as did CD4^+^ T-cell (5 trials; MD = 4.67, 95% CI: 1.30-8.05, *p* = 0.007; **Figure 6B**). In contrast, CD8^+^ T-cell levels decreased (5 trials; MD = -4.27, 95% CI: -5.34 to -3.19, *p* < 0.00001; **Figure 6C**). As a result, the CD4^+^/CD8^+^ ratio increased (5 trials; MD = 0.40, 95% CI: 0.24-0.57, *p* < 0.00001; **Figure 6D**).

### 3.9 Adverse events

#### 3.9.1 Hematological toxicities

**Figure 7:**
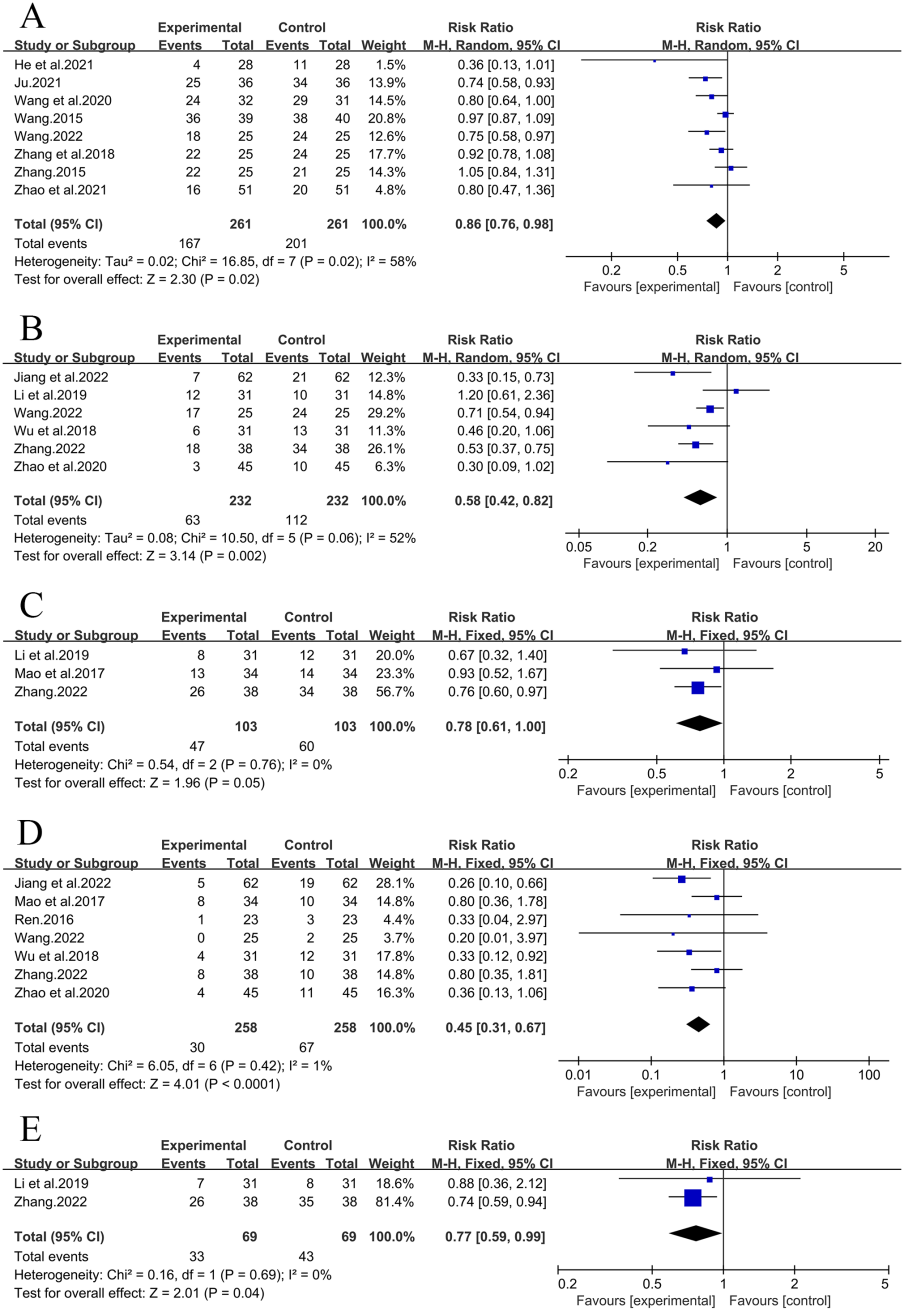
Forest plot of hematological toxicities. **(A)** Myelosuppression. **(B)** Leukopenia. **(C)** Neutropenia. **(D)** Thrombocytopenia. **(E)** Decreased hemoglobin.

Eight studies *(n* = 522) reported on myelosuppression were analyzed using a random-effects model. The combination therapy was associated with a lower risk of myelosuppression compared with chemotherapy alone (RR = 0.86, 95% CI: 0.76-0.98, *p* = 0.02, I² = 58%; **Figure 7A**). Six trials *(n* = 464) showed a reduced incidence of leukopenia in the combination group (RR = 0.58, 95% CI: 0.42-0.82, *p* = 0.002, I² = 52%; **Figure 7B**). Three trials (*n* = 206) were pooled using a fixed-effects model, showing a trend toward a reduction in neutropenia (RR = 0.78, 95% CI: 0.61-1.00, *p* = 0.05, I² = 0%; **Figure 7C**). Across six studies (*n* = 516), the occurrence of thrombocytopenia was lower in the combined treatment group (30 vs. 67 events). A fixed-effects model showed a significant reduction (RR = 0.45, 95% CI: 0.31-0.67, *p* < 0.0001, I² = 1%; **Figure 7D**). Finally, data from two trials (*n* = 138) analyzed via a fixed-effect model suggested a lower risk of decreased hemoglobin with combination therapy (RR = 0.77, 95% CI: 0.59-0.99, *p* = 0.04, I² = 0%; **Figure 7E**).

#### 3.9.2 Non-hematological toxicities

**Figure 8:**
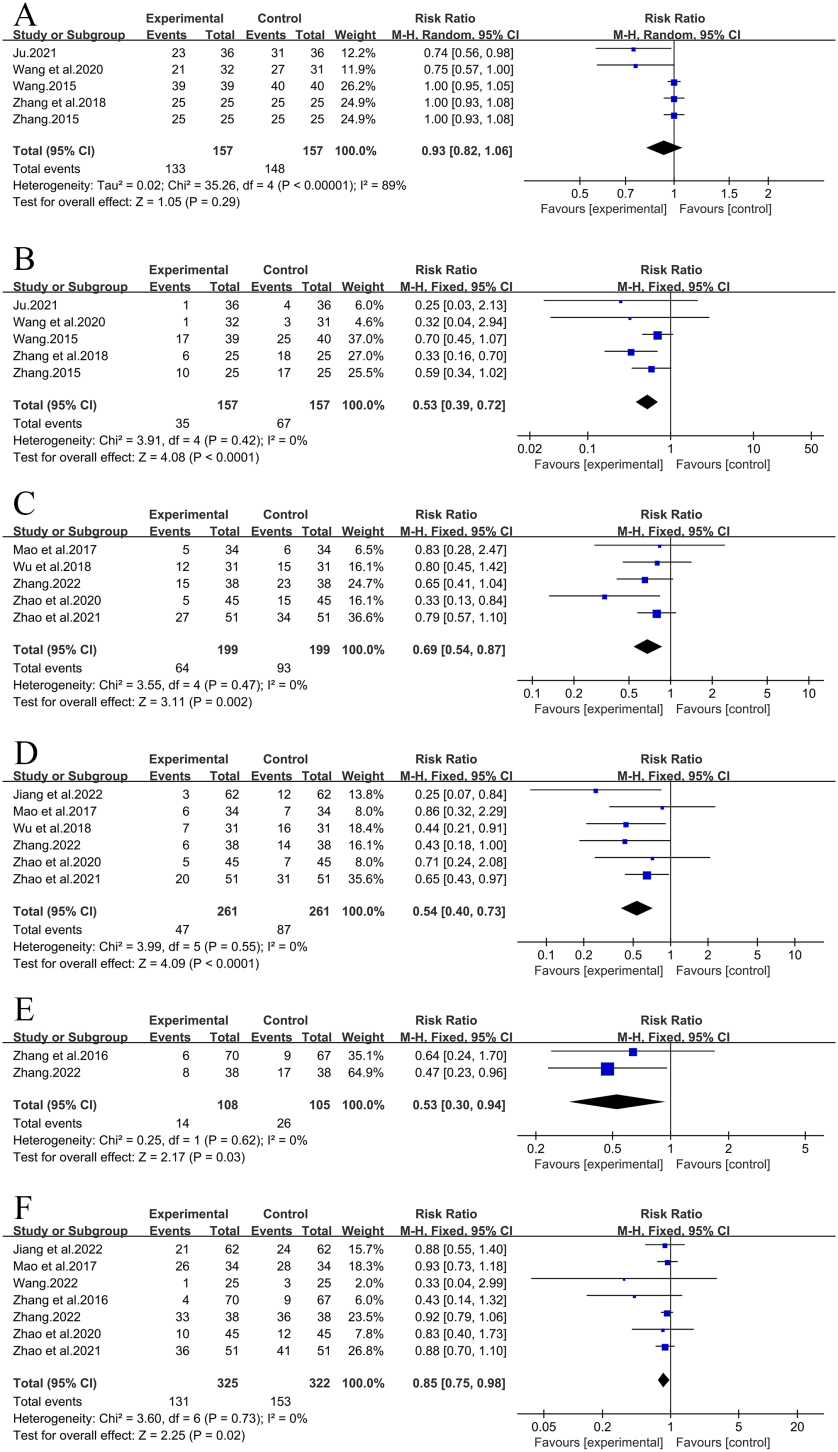
Forest plot of non-hematological toxicities. **(A)** Overall gastrointestinal reactions. **(B)** Severe (grade 3-4) gastrointestinal reactions. **(C)** Nausea. **(D)** Vomiting. **(E)** Diarrhea. **(F)** Alopecia.

Five studies (*n* = 314) reported overall gastrointestinal reaction and were analyzed using a random-effect model due to high heterogeneity (I² = 89%). No significant difference was observed between groups (RR = 0.93, 95% CI: 0.82-1.06, *p* = 0.29; **Figure 8A**). In contrast, fixed-effect model across 5 trials (*n* = 314) showed a lower incidence of for severe (grade III-IV) gastrointestinal reactions in the combination group (RR = 0.53, 95% CI: 0.39-0.72, *p* < 0.0001, I² = 0%; **Figure 8B**). Four studies (n = 398) showed a lower risk of nausea with combination therapy (RR = 0.69, 95% CI: 0.54-0.87, *p* = 0.002, I² = 0%; **Figure 8C**). Five studies (*n* = 522) reported vomiting, with a lower incidence in the combination group (RR = 0.54, 95% CI: 0.40-0.73, *p* < 0.0001, I² = 0%; **Figure 8D**). Two studies (*n* = 213) showed a reduction in diarrhea with combination therapy (RR = 0.53, 95% CI: 0.30-0.94, *p* = 0.03, I² = 0%; **Figure 8E**). Seven trials (*n* = 647) showed a lower risk of alopecia in the combination group (RR = 0.85, 95% CI: 0.75-0.98, *p* = 0.02, I² = 0%; **Figure 8F**).

### 3.10 Subgroup analysis

Subgroup analyses were conducted to examine whether treatment effects varied by clinical characteristics (TNM stage, chemotherapy regimens, and treatment duration) and by commonly used herbal components.

#### 3.10.1 Clinical characteristics

Detailed results are presented in **Table 2**. Patients were grouped into non-metastatic (TNM stages I-III) and metastatic (stage IV) disease. The combination therapy improved both ORR and DCR in each subgroup. There were no significant differences between subgroups (*p* = 0.68 for ORR; *p* = 0.82 for DCR), suggesting similar effects across disease stages. For chemotherapy regimens, studies were categorized into taxane-, platinum-, and anthracycline-based regimens. ORR and DCR improved in the taxane- and platinum-based subgroups. In the anthracycline-based subgroup, the improvement in ORR approached statistical significance (*p* = 0.07), while DCR did not reach significance (*p* = 0.13). As for treatment duration, studies were divided into ≤ 12 weeks and > 12 weeks. Both subgroups showed improvements in ORR and DCR. The >12-week subgroup showed a greater improvement in ORR, with a significant difference between subgroup (*p* = 0.005). No significant difference was observed for DCR (*p* = 0.47).

**Table 2:** Subgroup analysis of objective response rate and disease control rate.

Subgroups	Number of studies	RR(95%CI)	Z	P	Heterogeneity(I2)	Ph(Subgroup difference)
1. Objective response rate (ORR)						
Staging						0.68
Non-metastatic (Stage I-III)	5	1.38[1.05, 1.80]	2.34	0.02	70%	–
Metastatic (Stage IV)	14	1.29[1.15, 1.45]	4.3	<0.0001	7%	–
Chemotherapy regimen						0.48
Taxane-based	10	1.33[1.15, 1.54]	3.45	0.0005	56%	–
Platinum-based	4	1.29[1.04, 1.60]	2.23	0.02	0%	–
Anthracycline-containing	3	1.25[0.99, 1.58]	1.92	0.07	48%	–
Medication duration						0.005
≤12W	7	1.17[1.03, 1.33]	2.42	0.02	0%	–
>12W	10	1.53[1.33, 1.76]	5.98	<0.00001	40%	–
2. Disease control rate (DCR)						
Staging						0.82
Non-metastatic (Stage I-III)	5	1.16[1.06, 1.27]	3.33	0.001	9%	–
Metastatic (Stage IV)	14	1.15[1.08, 1.22]	4	<0.0001	0%	–
Chemotherapy regimen						0.48
Taxane-based	10	1.14[1.07, 1.23]	3.73	0.0002	0%	–
Platinum-based	4	1.23[1.08, 1.39]	3.33	0.001	0%	–
Anthracycline-containing	3	1.08[0.98, 1.20]	1.53	0.13	0%	–
Medication duration						0.47
≤12W	7	1.12[1.03, 1.21]	2.84	0.005	0%	–
>12W	10	1.16[1.08, 1.25]	4.17	<0.0001	0%	–

#### 3.10.2 Pattern analysis of high-frequency herb combinations

This analysis focused specifically on the 19 trials that provided both ORR and DCR data. As shown in **Table 3**, the herbs most frequently combined with Bupleurum were Radix Paeoniae Alba (n=17) and Poria (n=16), followed by Radix Salviae Miltiorrhizae (n=15), Radix Angelicae Sinensis (n=14), and Radix Astragali (n=12).

**Table 3:** Pattern analysis of high-frequency herb combinations for objective response rate and disease control rate.

Herb pair (latin name)	No.of Studies	ORR RR (95%CI)	ORR P-value	ORR I2	DCR RR (95%CI)	DCR P-value	DCR I2
Bupleurum + Radix Astragali (Huangqi)	12	1.42(1.22, 1.65)	<0.00001	34%	1.16(1.09, 1.24)	<0.00001	0%
Bupleurum + Poria (Fuling)	16	1.35(1.18, 1.55)	<0.0001	46%	1.14(1.08, 1.21)	<0.00001	0%
Bupleurum + Radix Angelicae Sinensis (Danggui)	14	1.30(1.14, 1.48)	<0.0001	37%	1.15(1.08, 1.21)	<0.00001	0%
Bupleurum + Radix Paeoniae Alba (Baishao)	17	1.30(1.16, 1.46)	<0.00001	33%	1.14(1.09, 1.20)	<0.00001	0%
Bupleurum + Rhizoma Atractylodis Macrocephalae (Baizhu)	15	1.25(1.13, 1.40)	<0.0001	16%	1.12(1.06, 1.19)	<0.0001	0%

It is important to note that this subgroup analysis of herbal pairings is strictly exploratory. Because the included trials utilized complex multi-herb formulas, the trial data overlapped across different subgroups, making them statistically non-independent. Consequently, these pooled effect sizes cannot be validly compared against each other or the overall estimate; rather, they should be viewed solely as descriptive trends.

For ORR, the exploratory data showed that formulas pairing Bupleurum with Radix Astragali yielded a relative risk (RR) of 1.42 (95% CI: 1.22-1.65, p < 0.00001), while combinations with Poria yielded an RR of 1.35 (95% CI: 1.18-1.55, p < 0.0001). Subgroups involving Radix Angelicae Sinensis and Radix Paeoniae Alba both showed an RR of 1.30 (95% CIs: 1.14-1.48 and 1.16-1.46, respectively; both p < 0.0001). For combinations containing Rhizoma Atractylodis Macrocephalae, the pooled RR was 1.25 (95% CI: 1.13-1.40; p < 0.0001). Regarding DCR, the descriptive effect estimates across these various combinations narrowly ranged from 1.12 to 1.16.

### 3.11 Sensitivity analysis

Sensitivity analyses were conducted by sequential excluding each study and recalculating the pooled estimates for ORR, DCR, and QOL. The results remained stable across all iterations, with no meaningful changes in effect size or statistical significance. This suggests that the overall findings were not driven by any single study.

### 3.12 Publication bias

Publication bias was assessed for outcomes with at least 10 studies (ORR and DCR) using funnel plots and performed Egger’s tests. For DCR, Egger’s test did not indicate significant publication bias (*p* = 0.135), and the funnel plot appeared symmetrical. For ORR, Egger’s test suggested potential publication bias (*p* = 0.004). After adjustment using the trim-and-fill method, with one study imputed, the pooled effect remained significant (RR = 1.36, 95% CI: 1.13-1.64), indicating that the main results was unchanged. For other outcomes (e.g., KPS and AEs), the number of studies was fewer than 10, and formal tests for publication bias were not performed in line with Cochrane guidance. Corresponding funnel plots are provided in the **Supplementary Material** (**Supplementary Figures 1**, **2**).

### 3.13 Certainty of evidence

The certainty of evidence was assessed using the GRADE approach (**Supplementary Table 2**). Evidence was rated as moderate for DCR and 1-year survival, as well as for severe (grade III-IV) gastrointestinal toxicity and specific AEs (including neutropenia, thrombocytopenia, decreased hemoglobin, nausea, vomiting, diarrhea, and alopecia). These outcomes were primarily downgraded by one level due to high trial-related bias, specifically the lack of allocation concealment and blinding, which may introduce subjective bias. Evidence for ORR was downgraded to low certainty due to a combination of high trial-related bias (lack of blinding) and significant publication bias (Egger’s test p=0.004). Evidence for PFS, QOL (KPS), tumor markers, immune function indicators, myelosuppression, leukopenia, and overall gastrointestinal toxicity was also rated as low certainty. These itemized downgrades were driven by high trial-related bias combined with significant clinical and statistical heterogeneity (I^2^ > 50%) across the included studies.

## 4 Discussion

According to the GLOBOCAN 2022 estimates, breast cancer remains the most common malignancy among women worldwide and the leading cause of cancer-related mortality in this population (42). In China, it is also a primary source of cancer burden (43). Among its molecular subtypes, TNBC is particularly challenging to manage due to its aggressive behavior, higher recurrence risk, and limited treatment options (44). Although immunotherapy and targeted therapies have expanded the treatment landscape in recent years, chemotherapy remains the backbone of TNBC treatment. However, its clinical benefit is often constrained by toxicity, which can lead to dose reduction or treatment discontinuation (45). In this context, combining TCM with chemotherapy has been explored as a way to improve tolerance and outcomes. Bupleurum-based formulas are widely used in clinical practice for this purpose. Preclinical studies suggest that active components of Bupleurum, particularly saikosaponins, have anti-tumor and immunomodulatory effects (46). However, clinical evidence has been fragmented.

This study synthesized available randomized evidence and found that Bupleurum-containing formulas combined with chemotherapy improved ORR and DCR in patients with TNBC. These findings are consistent with prior work (47) and extent earlier reviews of traditional East Asian medicine in breast cancer (48). Improvements were also observed in PFS and QOL, suggesting that the combination may enhance treatment response while supporting patient functioning during therapy.

Subgroup analyses indicated that treatment duration may play an important role. While benefits were observed in shorter interventions, studies with treatment duration greater than 12 weeks showed larger improvement in ORR. This pattern is consistent with the idea that the effects of Bupleurum-containing formulas may accumulate over time, potentially through gradual changes in the TME (49). Treatment effects were also similar across disease stages and across commonly used chemotherapy regimens, suggesting that the combination approach may be broadly applicable. Reduction in tumor markers (CEA, CA125, and CA15-3) were consistent with imaging-based measures of tumor response. Together, these findings support the overall pattern observed in pooled analysis, although they should be interpreted alongside the methodological limitations of the included studies.

Given the variation in TCM formulations, we conducted an exploratory analysis of commonly used herb combinations. Bupleurum was frequently paired with Radix Astragali, showing a favorable trend in tumor response. However, due to overlapping primary trials, these subgroup analyses are strictly exploratory and cannot establish a definitive efficacy ranking among combinations. One possible explanation for this potential trend is the complementary roles attributed to these herbs in TCM: Bupleurum is often used for its direct anti-tumor activity, whereas Astragalus is used to support host function. Experimental studies suggest that Astragalus polysaccharides can influence immune pathways and hematopoiesis (50–52), which may relate to the observed reductions in hematological toxicities. By mitigating these toxicities, this traditional “Fu Zheng Qu Xie” (strengthening body resistance to eliminate pathogens) strategy may help patients maintain the prescribed chemotherapy dose intensity, a key driver of improved survival in TNBC (53). These mechanisms remain speculative in the context of the present analysis and require further validation. Although our analysis covered these combined herbs, differences in preparation methods can significantly alter pharmacokinetics. In our current pool, traditional decoctions overwhelmingly dominated, with only one study [Song 2018 (29)] utilizing a capsule formulation. Consequently, meaningful subgroup comparisons based on dosage forms are currently unfeasible, highlighting the critical need for standardized herbal preparations in future trials.

At the molecular level, several mechanisms have been proposed for Bupleurum-derived compounds. Saikosaponin D has been shown to inhibit tumor growth through pathways involving STAT3 and c-Myc, and to induce apoptosis through effects on autophagy-related processes (54, 55). It may also enhance the effects of chemotherapy by altering intracellular redox balance and interacting with tumor necrosis factor–related pathways (56, 57). Saikosaponin A has been associated with inhibition of the PI3K/Akt pathway and may reduce metastatic potential (58, 59). While these findings provide biological plausibility, their clinical relevance remains to be established.

Consistent with these mechanisms, the present analysis found that combination therapy was associated with changes in immune markers, including increases in CD3^+^ and CD4^+^ T cells and in the CD4^+^/CD8^+^ ratio. This transition of the TME from a “cold” to a “hot” state aligns with the pharmacological actions of Bupleurum polysaccharides and saikosaponins in promoting lymphocyte activation (60, 61). Safety findings also suggest a reduction in several chemotherapy-related adverse events, including myelosuppression and severe gastrointestinal toxicity. These effects may contribute to improved treatment tolerance, although the underlying mechanisms may be associated with the regulation of the brain-gut axis and gastrointestinal motility (62) but are not fully understood. Finally, the combined treatment reduced the risk of alopecia, which is of great significance in alleviating psychological distress in female breast cancer patients (63).

This study has the following limitations. Firstly, we only searched for literature in Chinese and English databases, and all included RCTs were exclusively performed in China. This single-region recruitment significantly limits the external validity of our findings. Tumor biology, particularly the distribution of TNBC molecular subtypes and genomic mutational landscapes, exhibits certain differences between East Asian cohorts and Western populations (64, 65). Furthermore, environmental and dietary differences strongly shape gut microbiota composition, which may influence the pharmacokinetics of oral traditional Chinese medicine. Additionally (66, 67), Egger’s test reflected potential publication bias for ORR, although it was adjusted using the trim-and-fill method. Secondly, in most of the included trials, allocation concealment and blinding were “unclear”. The absence of blinding introduces subjective bias toward efficacy and adverse event adjudication, leading to the downgraded quality of outcomes based on the GRADE approach. Thirdly, there was substantial clinical heterogeneity among studies due to inconsistent herbal dosages, processing methods, and administration frequencies, as well as disparate evaluation criteria for QOL and adverse events. This might have weakened the evidence strength of this meta-analysis. Fourthly, Bupleurum is rarely used as a monotherapy in the treatment of TNBC; it is usually combined with other herbs. The sparse reporting of specific safety indicators within these small sub-cohorts currently restricts a comprehensive statistical evaluation of adverse events for different herbal pairings. And lastly, most of the trials incorporated might not have been reported strictly according to the CONSORT reporting criteria. All these limitations may have led to an inadequate assessment of certain outcomes. However, we hope these findings will encourage further research and help clinicians better understand the potential role of Bupleurum-containing TCM in managing TNBC.

## 5 Conclusion

Bupleurum-containing formulas combined with chemotherapy appear to be associated with improved clinical outcomes and reduced AEs in Chinese (and broadly East Asian) patients with TNBC. However, these findings should be interpreted with caution due to the low-to-moderate certainty of the current evidence, and further high-quality research is required to confirm these results.

## Data Availability

The original contributions presented in the study are included in the article/**Supplementary Material**. Further inquiries can be directed to the corresponding authors.
