# Supplementary material for: Efficacy and safety of bupleurum-containing formulas combined with chemotherapy for triple-negative breast cancer: a systematic review and meta-analysis
Source: Front Oncol. 2026 Jul 15;16:1867349. doi: 10.3389/fonc.2026.1867349 (PMC13414758; doi:10.3389/fonc.2026.1867349)
Supplement: Supplementary file 1 [file DataSheet1.pdf]

Supplementary Material

1 Supplementary Data

Search Strategy

pubmed:
("Triple Negative Breast Neoplasms"[Mesh] OR "Triple negative breast cancer"[Title/Abstract] OR "TNBC"[Title/Abstract] OR "Triple-negative breast carcinoma"[Title/Abstract] OR "Basal-like breast cancer"[Title/Abstract])
AND
("Bupleurum"[Mesh] OR "Drugs, Chinese Herbal"[Mesh] OR "Medicine, Chinese Traditional"[Mesh] OR "Chaihu"[Title/Abstract] OR "Radix Bupleuri"[Title/Abstract] OR "Bupleurum"[Title/Abstract] OR "Saikosaponin"[Title/Abstract] OR "Xiao Chaihu"[Title/Abstract] OR "Xiaoyao"[Title/Abstract] OR "Sini San"[Title/Abstract] OR "Shugan"[Title/Abstract] OR "Jianpi"[Title/Abstract] OR "Liqi"[Title/Abstract] OR "Jieyu"[Title/Abstract] OR "Soothing liver"[Title/Abstract] OR "Invigorating spleen"[Title/Abstract])
AND
("Drug Therapy"[Mesh] OR "Antineoplastic Combined Chemotherapy Protocols"[Mesh] OR "Chemotherapy"[Title/Abstract] OR "Adjuvant chemotherapy"[Title/Abstract] OR "Neoadjuvant chemotherapy"[Title/Abstract] OR "Taxane"[Title/Abstract] OR "Anthracycline"[Title/Abstract] OR "Platinum"[Title/Abstract] OR "Capecitabine"[Title/Abstract])
AND
("Randomized Controlled Trial"[Publication Type] OR "Randomized Controlled Trials as Topic"[Mesh] OR "randomized"[Title/Abstract] OR "placebo"[Title/Abstract] OR "randomly"[Title/Abstract])

Embase:
('triple negative breast cancer'/exp OR 'triple negative breast cancer':ti,ab,kw OR 'tnbc':ti,ab,kw)
AND
('bupleurum'/exp OR 'chinese herbal drug'/exp OR 'traditional chinese medicine'/exp OR 'chaihu':ti,ab,kw OR 'radix bupleuri':ti,ab,kw OR 'bupleurum':ti,ab,kw OR 'saikosaponin':ti,ab,kw OR 'xiao chaihu':ti,ab,kw OR 'chaihu shugan':ti,ab,kw OR 'xiaoyao':ti,ab,kw OR 'shugan':ti,ab,kw OR 'jianpi':ti,ab,kw OR 'liqi':ti,ab,kw OR 'jieyu':ti,ab,kw)
AND
('chemotherapy'/exp OR 'antineoplastic agent'/exp OR 'chemotherapy':ti,ab,kw OR 'adjuvant chemotherapy':ti,ab,kw OR 'neoadjuvant chemotherapy':ti,ab,kw)
AND
('randomized controlled trial'/exp OR 'randomization'/exp OR 'random*':ti,ab,kw)

Cochrane
#1,MeSH descriptor: [Triple Negative Breast Neoplasms] explode all trees
#2,"(""triple negative breast cancer"" OR ""TNBC"" OR ""triple-negative breast"" OR ""basal-like breast cancer""):ti,ab,kw"
#3,#1 OR #2
#4,MeSH descriptor: [Bupleurum] explode all trees
#5,"MeSH descriptor: [Drugs, Chinese Herbal] explode all trees"
#6,"(""Chaihu"" OR ""Radix Bupleuri"" OR ""Bupleurum"" OR ""Xiao Chaihu"" OR ""Xiaoyao"" OR ""Shugan"" OR ""Jianpi"" OR ""Liqi"" OR ""Jieyu"" OR ""Chinese herbal""):ti,ab,kw"
#7,#4 OR #5 OR #6
#8,MeSH descriptor: [Antineoplastic Agents] explode all trees
#9,"(""Chemotherapy"" OR ""Chemo"" OR ""Adjuvant chemotherapy"" OR ""Neoadjuvant chemotherapy"" OR ""Taxane"" OR ""Anthracycline"" OR ""Platinum""):ti,ab,kw"
#10,#8 OR #9
#11,#3 AND #7 AND #10

中国知网 (CNKI)

SU=("三阴性乳腺癌" + "三阴乳腺癌" + "三阴性乳癌" + "三阴性乳岩" + "TNBC") * SU=("柴胡" + "疏肝" + "解郁" + "理气" + "小柴胡" + "逍遥" + "四逆散" + "柴胡疏肝" + "中药" + "复方") * SU=("化疗" + "化学治疗" + "新辅助化疗" + "辅助化疗" + "紫杉醇" + "蒽环" + "铂类" + "卡培他滨")

万方 (Wanfang)

(主题:("三阴性乳腺癌" OR "三阴乳腺癌" OR "三阴性乳癌" OR "三阴性乳岩" OR "TNBC")) AND (主题:("柴胡" OR "疏肝" OR "解郁" OR "理气" OR "小柴胡" OR "逍遥" OR "四逆散" OR "柴胡疏肝" OR "中药" OR "复方")) AND (主题:("化疗" OR "化学治疗" OR "新辅助化疗" OR "辅助化疗" OR "紫杉醇" OR "蒽环" OR "铂类" OR "卡培他滨"))

维普中文科技期刊数据库 (VIP)

(U="三阴性乳腺癌" OR U="三阴乳腺癌" OR U="三阴性乳癌" OR U="三阴性乳岩" OR U="TNBC") AND (U="柴胡" OR U="疏肝" OR U="解郁" OR U="理气" OR U="小柴胡" OR U="逍遥" OR U="四逆散" OR U="柴胡疏肝" OR U="中药" OR U="复方") AND (U="化疗" OR U="化学治疗" OR U="新辅助化疗" OR U="辅助化疗" OR U="紫杉醇" OR U="蒽环" OR U="铂类" OR U="卡培他滨")

中国生物医学文献数据库 (SinoMed)

("三阴性乳腺肿瘤"[主题词] OR "三阴性乳腺癌"[常用字段] OR "三阴乳腺癌"[常用字段] OR "三阴性乳癌"[常用字段] OR "三阴性乳岩"[常用字段] OR "TNBC"[常用字段]) AND ("柴胡"[主题词] OR "柴胡"[常用字段] OR "疏肝"[常用字段] OR "解郁"[常用字段] OR "理气"[常用字段] OR "小柴胡"[常用字段] OR "逍遥"[常用字段] OR "四逆散"[常用字段] OR "中药"[常用字段] OR "复方"[常用

字段]) AND ("抗肿瘤联合化疗方案"[主题词] OR "化疗"[常用字段] OR "化学治疗"[常用字段] OR "新辅助化疗"[常用字段] OR "紫杉醇"[常用字段] OR "铂类"[常用字段])

2 Supplementary Figures and Tables

2.1 Supplementary Figures

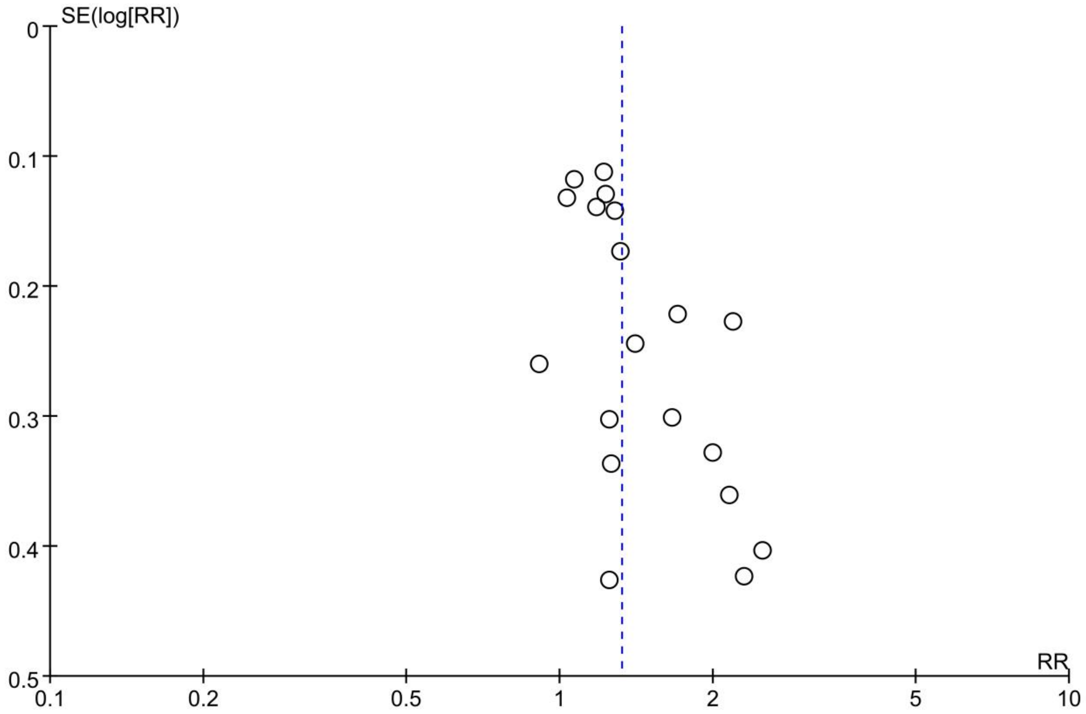

Supplementary Figure S1. ORR funnel plot

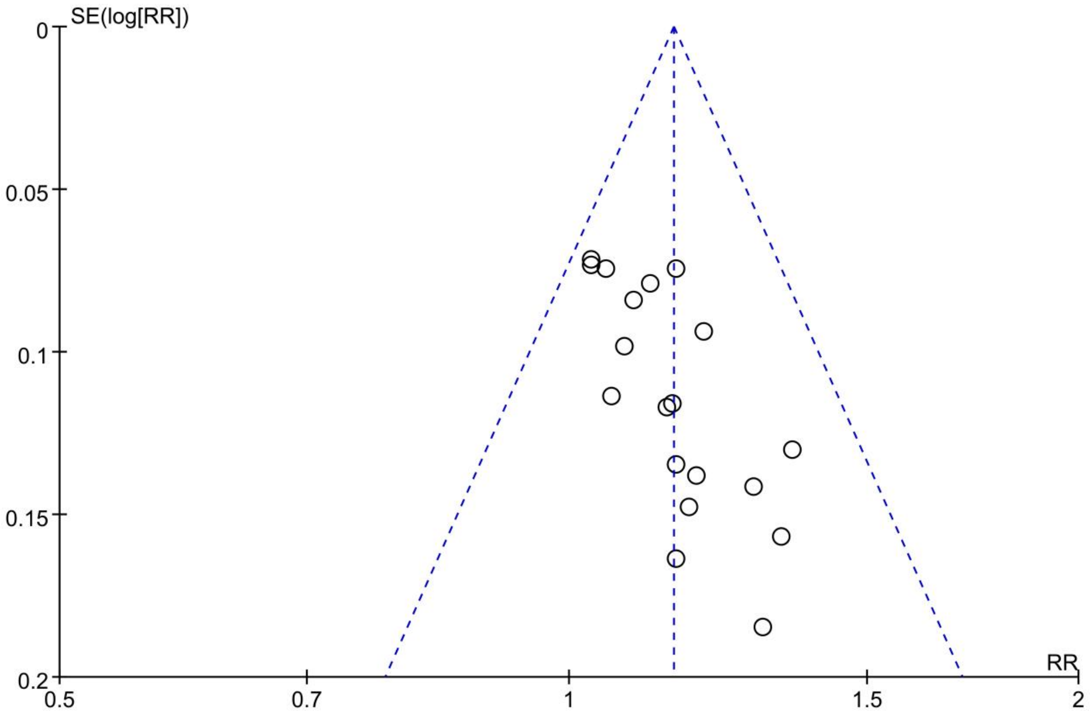

Supplementary Figure S2. DCR funnel plot

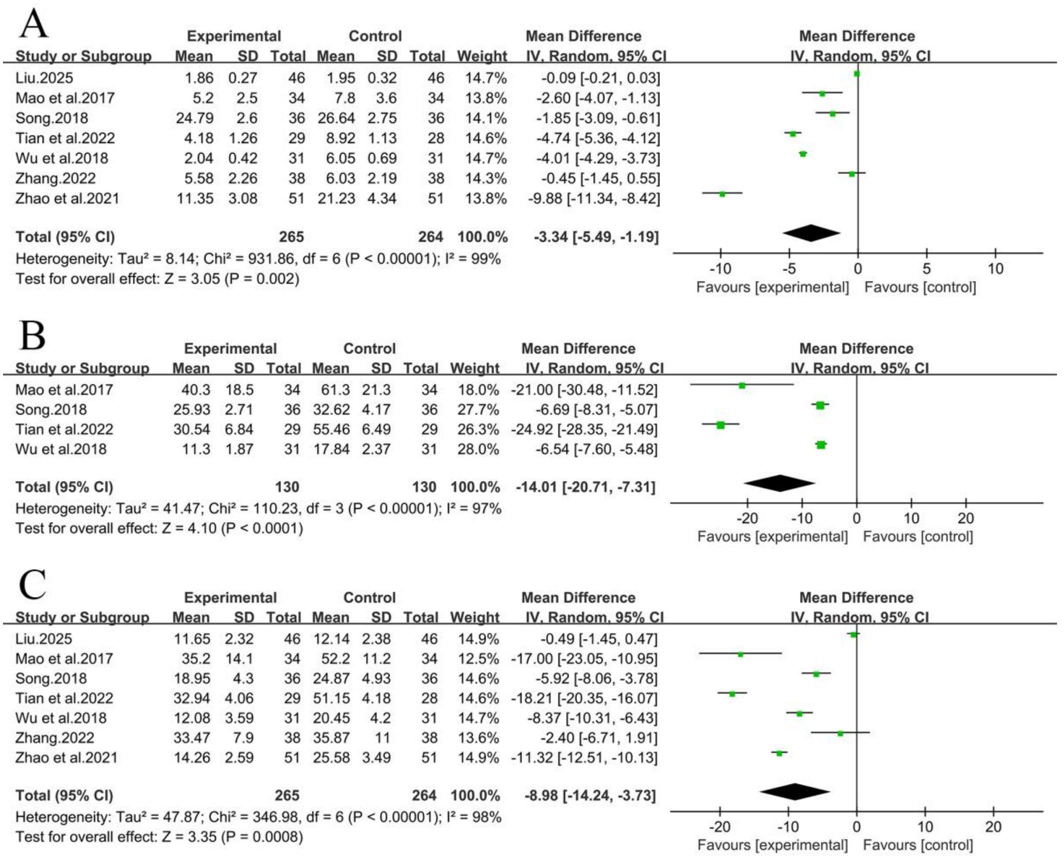

Supplementary Figure S3. Tumor Markers funnel plot
(A) CEA. (B) CA125. (C) CA15-3.

2.2 Supplementary Tables

Supplementary Table S1: Compositions and detailed herbal ingredients of the Traditional Chinese Medicine (TCM) formulas in the included studies.

Study ID	Formula Name	Detailed Composition (Latin Name Only)
**Cui.2015**	Chaihu-based Formula	Radix Bupleuri; Radix Paeoniae Alba; Radix Curcumae; Rhizoma Cyperi; Radix Codonopsis; Rhizoma Atractylodis Macrocephalae; Poria; Semen Coicis; Spica Prunellae; Herba Hedyotis Diffusae.
**He et al.2021**	Cigu Pingyan Decoction	Radix Bupleuri; Bulbus Cremastrae; Radix Astragali; Radix Codonopsis; Radix Curcumae; Polyporus; Poria; Rhizoma Curcumae; Rhizoma Sparganii; Spina Gleditsiae.
**Jiang et al.2022**	Shugan Yishen Formula	Radix Bupleuri; Radix Astragali; Radix Paeoniae Alba; Rhizoma Atractylodis Macrocephalae; Rhizoma Dioscoreae; Sargassum; Herba Hedyotis Diffusae; Radix Arnebiae; Bulbus Cremastrae.
**Ju.2021**	Shugan Jianpi Formula	Radix Bupleuri; Radix Pseudostellariae; Fructus Akebiae; Radix Paeoniae Alba; Poria; Rhizoma Atractylodis Macrocephalae; Flos Chrysanthemi; Radix Glycyrrhizae.
**Li et al.2019**	Chaihu-based Formula	Radix Bupleuri; Radix Astragali; Radix Curcumae; Rhizoma Atractylodis Macrocephalae; Radix Codonopsis; Poria; Radix Angelicae Sinensis; Rhizoma Dioscoreae; Radix Paeoniae Alba.
**Li.2014**	Chaihu-based Formula	Radix Bupleuri; Radix Paeoniae Alba; Radix Ginseng; Rhizoma Atractylodis Macrocephalae; Poria; Pericarpium Citri Reticulatae; Bulbus Fritillariae Thunbergii; Rhizoma Pinelliae; Cortex Magnoliae Officinalis.
**Lin.2016**	Chaihu-based Formula	Radix Bupleuri; Radix Rehmanniae Praeparata; Radix Angelicae Sinensis; Radix Paeoniae Alba; Poria; Rhizoma Atractylodis Macrocephalae; Rhizoma Dioscoreae; Pericarpium Citri Reticulatae.
**Liu.2025**	Shugan Jieyu Decoction	Radix Bupleuri; Herba Hedyotis Diffusae; Fructus Aurantii; Radix Paeoniae Alba; Radix Glycyrrhizae; Pericarpium Citri Reticulatae; Rhizoma Chuanxiong; Rhizoma Cyperi.
**Mao et al.2017**	Fuzheng Hualiu Formula	Radix Bupleuri; Radix Codonopsis; Radix Astragali; Herba Hedyotis Diffusae; Radix Angelicae Sinensis; Rhizoma Dioscoreae; Pericarpium Citri Reticulatae Viride.
**Ren.2016**	Chaihu-based Formula	Radix Bupleuri; Radix Rehmanniae Praeparata; Radix Astragali; Radix Angelicae Sinensis; Rhizoma Atractylodis

		Macrocephalae; Radix Paeoniae Alba; Colla Cornus Cervi; Poria.
**Song.2018**	Shugan Huazheng Capsule	Radix Bupleuri; Carapax Trionycis; Bulbus Fritillariae; Radix Angelicae Sinensis; Radix Paeoniae Alba; Herba Scutellariae Barbatae; Poria; Rhizoma Atractylodis Macrocephalae; Radix Glycyrrhizae.
**Tian et al.2022**	Shugan Liqi Xiaoyan Formula	Radix Bupleuri; Fructus Citri Sarcodactylis; Fructus Citri; Radix Curcumae; Radix Codonopsis; Rhizoma Atractylodis Macrocephalae; Fructus Aurantii; Fructus Schisandrae.
**Wang et al.2020**	Shugan Jianpi Formula	Radix Bupleuri; Radix Pseudostellariae; Radix Paeoniae Alba; Fructus Akebiae; Flos Chrysanthemi; Rhizoma Atractylodis Macrocephalae; Poria.
**Wang.2015**	Chaihu-based Formula	Radix Bupleuri; Radix Rehmanniae Praeparata; Radix Astragali; Colla Cornus Cervi; Radix Angelicae Sinensis; Radix Paeoniae Alba; Rhizoma Atractylodis Macrocephalae; Poria.
**Wang.2022**	Shugan Jianpi Yishen Decoction	Radix Bupleuri; Radix Astragali; Radix Rehmanniae Praeparata; Colla Cornus Cervi; Radix Angelicae Sinensis; Radix Paeoniae Alba; Rhizoma Atractylodis Macrocephalae.
**Wu et al.2018**	Chaihu-based Formula	Radix Bupleuri; Radix Rehmanniae Praeparata; Radix Astragali; Colla Cornus Cervi; Endothelium Corneum Gigeriae Galli; Fructus Corni; Rhizoma Dioscoreae; Radix Angelicae Sinensis.
**Zhang et al.2016**	Pingyu Shiru Formula	Radix Bupleuri; Rhizoma Cyperi; Fructus Trichosanthis; Herba Hedyotis Diffusae; Caulis Spatholobi; Bulbus Cremastrae; Radix Glycyrrhizae.
**Zhang et al.2018**	Tiaopi Shugan Yishen Formula	Radix Bupleuri; Radix Rehmanniae Praeparata; Radix Astragali; Colla Cornus Cervi; Rhizoma Zingiberis Recens; Fructus Corni; Radix Paeoniae Alba.
**Zhang.2015**	Chaihu-based Formula	Radix Bupleuri; Radix Rehmanniae Praeparata; Radix Astragali; Colla Cornus Cervi; Radix Angelicae Sinensis; Radix Paeoniae Alba; Rhizoma Atractylodis Macrocephalae.
**Zhang.2022**	Xiaoyao Loubei Formula	Radix Bupleuri; Concha Ostreae; Fructus Trichosanthis; Bulbus Fritillariae Thunbergii; Poria; Rhizoma Pinelliae Preparata; Radix Paeoniae Alba.
**Zhao et al.2020**	Shugan Jianpi Yishen Decoction	Radix Bupleuri; Radix Rehmanniae Praeparata; Radix Astragali; Colla Cornus Cervi; Radix Angelicae Sinensis; Radix Paeoniae Alba; Rhizoma Atractylodis Macrocephalae.
**Zhao.et al.2021**	Shugan Jianpi Jiangni Formula	Radix Bupleuri; Radix Codonopsis; Radix Astragali; Fructus Ligustri Lucidi; Poria; Radix Polygoni Multiflori; Radix Angelicae Sinensis.

**Zhu.2014**	Xiaoyao San	Radix Bupleuri; Radix Angelicae Sinensis; Radix Paeoniae Alba; Poria; Rhizoma Atractylodis Macrocephalae; Radix Glycyrrhizae.

Supplementary Table S2: GRADE summary of findings and certainty of evidence.

Outcomes	№ of studies	Design	Risk of bias	Inconsistency	Indirectness	Imprecision	Other	Patients (Int / Ctrl)	Relative Effect (95% CI)	Absolute Effect (95% CI)	Certainty
**1. Efficacy Outcomes**											
ORR	19	RCTs	Serious[a]	Not serious	Not serious	Not serious	Suspected[b]	408/636 vs 295/630	RR 1.32 (1.18 to 1.48)	150 more per 1,000	⊕⊕◯◯ Low
DCR	19	RCTs	Serious[a]	Not serious	Not serious	Not serious	None	561/636 vs 483/630	RR 1.15 (1.09 to 1.21)	115 more per 1,000	⊕⊕⊕◯ Moderate
1-year Survival	3	RCTs	Serious[a]	Not serious	Not serious	Not serious	None	120/134 vs 100/128	RR 1.15 (1.03 to 1.28)	117 more per 1,000	⊕⊕⊕◯ Moderate
PFS	3	RCTs	Serious[a]	Serious[c]	Not serious	Not serious	None	102 vs 104	-	MD 2.36 higher	⊕⊕◯◯ Low
KPS Score	9	RCTs	Serious[a]	Serious[c]	Not serious	Not serious	None	329 vs 321	-	MD 6.48 higher	⊕⊕◯◯ Low
**2. Tumor Markers**											
CEA	7	RCTs	Serious[a]	Serious[c]	Not serious	Not serious	None	265 vs 264	-	MD 3.34 lower	⊕⊕◯◯ Low
CA125	4	RCTs	Serious[a]	Serious[c]	Not serious	Not serious	None	130 vs 130	-	MD 14.01 lower	⊕⊕◯◯ Low
CA15-3	7	RCTs	Serious[a]	Serious[c]	Not serious	Not serious	None	265 vs 264	-	MD 8.98 lower	⊕⊕◯◯ Low
**3. Immune Function**											
$CD3^+$ T	4	R	Seri	Serio	Not	Not	No	138 vs	-	MD 9.06	⊕⊕◯◯

cells		RCTs	Serious[a]	Serious[c]	Not serious	Not serious	None	138		higher	Low
CD4+ T cells	5	RCTs	Serious[a]	Serious[c]	Not serious	Not serious	None	184 vs 184	-	MD 4.67 higher	⊕⊕◯◯ Low
CD8+ T cells	5	RCTs	Serious[a]	Serious[c]	Not serious	Not serious	None	184 vs 184	-	MD 4.27 lower	⊕⊕◯◯ Low
CD4+/CD8+ Ratio	5	RCTs	Serious[a]	Serious[c]	Not serious	Not serious	None	184 vs 184	-	MD 0.40 higher	⊕⊕◯◯ Low
**4. Safety (Hematological)**											
Myelosuppression	8	RCTs	Serious[a]	Serious[c]	Not serious	Not serious	None	167/261 vs 201/261	RR 0.86 (0.76 to 0.98)	108 fewer per 1,000	⊕⊕◯◯ Low
Leukopenia	6	RCTs	Serious[a]	Serious[c]	Not serious	Not serious	None	63/232 vs 112/232	RR 0.58 (0.42 to 0.82)	203 fewer per 1,000	⊕⊕◯◯ Low
Neutropenia	3	RCTs	Serious[a]	Not serious	Not serious	Not serious	None	47/103 vs 60/103	RR 0.78 (0.61 to 1.00)	128 fewer per 1,000	⊕⊕⊕◯ Moderate
Thrombocytopenia	7	RCTs	Serious[a]	Not serious	Not serious	Not serious	None	30/258 vs 67/258	RR 0.49 (0.33 to 0.72)	132 fewer per 1,000	⊕⊕⊕◯ Moderate
Hemoglobin	2	RCTs	Serious[a]	Not serious	Not serious	Not serious	None	33/69 vs 43/69	RR 0.77 (0.59 to 0.99)	143 fewer per 1,000	⊕⊕⊕◯ Moderate
**5. Safety (Gastrointestinal)**											
Overall GI Toxicity	5	RCTs	Serious[a]	Serious[c]	Not serious	Not serious	None	133/157 vs 148/157	RR 0.93 (0.82 to 1.06)	66 fewer per 1,000	⊕⊕◯◯ Low
Severe (Grade 3-4) GI Toxicity	5	RCTs	Serious[a]	Not serious	Not serious	Not serious	None	35/157 vs 67/157	RR 0.53 (0.39 to 0.72)	201 fewer per 1,000	⊕⊕⊕◯ Moderate
Nausea	5	RCTs	Serious[a]	Not serious	Not serious	Not serious	None	64/199 vs 93/199	RR 0.69 (0.54 to 0.87)	145 fewer per 1,000	⊕⊕⊕◯ Moderate

Vomiting	6	RCTs	Serious [a]	Not serious	Not serious	Not serious	None	47/261 vs 87/261	RR 0.54 (0.40 to 0.73)	153 fewer per 1,000	⊕⊕⊕◯ Moderate
Diarrhea	2	RCTs	Serious [a]	Not serious	Not serious	Not serious	None	14/108 vs 26/105	RR 0.53 (0.30 to 0.94)	116 fewer per 1,000	⊕⊕⊕◯ Moderate
Alopecia	7	RCTs	Serious [a]	Not serious	Not serious	Not serious	None	131/325 vs 153/322	RR 0.85 (0.75 to 0.98)	71 fewer per 1,000	⊕⊕⊕◯ Moderate

Abbreviations: RCTs, randomized controlled trials; Int, intervention group; Ctrl, control group; RR, risk ratio; MD, mean difference; CI, confidence interval; ORR, objective response rate; DCR, disease control rate; PFS, progression-free survival; KPS, Karnofsky Performance Status; CEA, carcinoembryonic antigen; GI, gastrointestinal. Footnotes: [a] Downgraded by 1 level for risk of bias: Most included primary trials failed to report allocation concealment and proper blinding schemes. This absence of blinding introduces subjective bias, particularly toward efficacy and adverse event adjudication. [b] Downgraded by 1 level for publication bias: Egger's test showed statistical significance ($P = 0.004$), indicating the presence of publication bias, likely driven by the preferential publication of positive results. [c] Downgraded by 1 level for inconsistency: High statistical heterogeneity ($I^2 > 50\%$) was observed among the included studies, and subgroup analyses could not fully explain the source of heterogeneity.
